# Effects of Intra- and Interpatch Host Density on Egg Parasitism by Three Species of *Trichogramma*

**DOI:** 10.1673/031.010.9901

**Published:** 2010-07-08

**Authors:** Matthew J. Grieshop, Paul W. Flinn, James R. Nechols

**Affiliations:** ^1^ Kansas State University, Department of Entomology, 123 West Waters Hall, Manhattan, KS66506-4004, USA; ^2^ USDA-ARS, Grain Marketing and Production Research Center, 1515 College Avenue, Manhattan Kansas 66502, USA; ^3^ Current address: Michigan State University, Department of Entomology, 205 Center for Integrated Plant Systems, East Lansing, MI 48824

**Keywords:** biological control, foraging behavior, *Plodia interpunctella*, spatial scale, stored products, *Trichogramma deion*, *Trichogramma ostriniae*, *Trichogramma pretiosum*

## Abstract

Host-foraging responses to different intra- and interpatch densities were used to assess three *Trichogramma* spp. (Hymenoptera: Trichogrammatidae) *Trichogramma deion* Pinto and Oatman, *T. ostriniae* Pang and Chen, and *T. pretiosum* Riley — as potential biological control agents for the Indian meal moth, *Plodia interpunctella* Hübner (Lepidoptera: Pyralidae). Single naïve females were allowed 6 h to forage in Plexiglas arenas with four different spatial arrangements of host eggs, nine single-egg patches), nine four-egg patches, 36 single-egg patches, and 36 four-egg patches. No significant differences were found among species in the number of patches parasitized. As expected, all three species parasitized the most eggs in the 36 four-egg patch treatment and the least in the nine single-egg patch treatment. *T. deion* parasitized significantly more eggs than *T. pretiosum* on the nine four-egg patches. *T. ostriniae* parasitized significantly more patches when intrapatch density was greater, regardless of interpatch density. In contrast, *T. deion* only parasitized more patches at the greater intrapatch density when the interpatch density was low. Patch density had no effect on *T. pretiosum*. The spatial pattern of parasitism was more aggregated for *T. deion* and *T. ostriniae* in the 36 four-egg patches treatment compared to the 36 single-egg patches treatment. Therefore, intrapatch density was more important than interpatch density for *T. ostriniae*, and potentially for *T. deion*, but not for *T. pretiosum. T. deion* may be the best candidate for augmentative biological control because it parasitized either slightly or significantly more eggs than the other two species in all four treatments. Furthermore, the pattern of parasitism by *T. deion* in the 36 four-egg patches treatment was the most aggregated among the three species, suggesting a more thorough searching pattern. In contrast, *T. pretiosum* had the least aggregated pattern of parasitism and therefore may have used a more random foraging pattern.

## Introduction

The Indian meal moth, *Plodia interpunctella* (Lepidoptera: Pyralidae) (Hübner), is a cosmopolitan pest of stored products found in bulk, processing, warehouse, and retail systems (Cox and Bell 1991). The range of products attacked by this pest includes: raw and finished cereals, nuts, dried fruit and vegetables, pulses, and garlic (Perez-Mendoza and Aguilera-Pena 2004). Moths cause damage by infesting both unprocessed, bulk products as well as finished, retail commodities. In bulk products, they are generally considered to be a nuisance pest. However, infestations in retail stores and warehouses result in significant damage, because the presence of a single larva or adult may result in the total loss of the infested product, as well as potential customer complaints or even lawsuits. The traditional management strategy for *P. interpunctella* has been the use of chemical insecticides, including: fog, fumigant, and grain protectants (Cox and Bell 1991). However, recent legislation has reduced the availability of chemical insecticides for use on stored products (Arthur and Rogers 2003).

*Trichogramma* (Hymenoptera: Trichogrammatidae) egg parasitoids have recently been studied as augmentative biological control agents for *P. interpunctella* in stored product systems, including: bulk peanut storage (Brower 1988), bulk wheat storage (Schöller et al. 1996), bakeries (Prozeil and Schöller 1998; Steidle et al. 2001), as well as in warehouses and retail stores (Schöller et al. 2006). *Trichogramma* spp. present some unique advantages that make them well-suited for use in the high visibility/low economic threshold systems characteristic of finished stored products. These include: causing mortality to the egg stage prior to the damaging larval stage, commercial availability, and perhaps most importantly, tiny body size (< 0.5 mm in length), making them unlikely to be noticed by consumers.

Selection of an appropriate species and/or strain of biological control agent is a critically important part of any augmentative biological control program (Smith 1996; Van Driesche and Bellows 1996), and has been discussed specifically in regards to choosing trichogrammatids to manage stored product moths (Schöller et al. 1994). The fact that *P. interpunctella*, unlike other pyralid moths such as *Ostrinia nubilalis*, lays its eggs both singly and in small clusters, and does so either directly on exposed products (Arbogast and Mullen 1978) or adjacent to compromised packages in response to food odors (Mullen 1994), results in a heterogeneous distribution of small (< 10) localized patches of host eggs. In general, variation in intra- and interpatch host density is known to affect parasitoid foraging behavior and success (Hassell 1982). In studies of *Trichogramma* spp., increased numbers of host eggs within patches have been shown to have a positive effect on host patch location (Morrison et al. 1980; Kfir 1983; Reznik and Umarova 1991; McCravy and Berisford 1998; Barnay et al. 1999) and foraging activity (Nordlund et al. 1981).

Therefore, the effects of four intra- and interpatch densities of *P. interpunctella* eggs were examined on the host-foraging success of individual naïve females of three *Trichogramma* species. The experimental objective was to select the most suitable species for use in an augmentative biological control program for this pest in retail stores and warehouses. Experimental assessments included measurement of overall parasitism, as well as a simple assessment of the relative aggregation of parasitism between two intrapatch host densities. The latter assessment was perceived as important relative to the management of *P. interpunctella* due to this pest's tendency to lay single or small clusters of eggs adjacent to compromised food packages or product spills (Arbograst and Mullen 1978; Mullen 1994), which may result in a concentration of eggs within the vicinity of such host resources. Thus, a more aggregated pattern of parasitism is likely to be desirable, as it would lead to a more thorough exploitation of areas likely to contain hosts. This experiment was performed to complement additional studies evaluating the foraging efficiency of the same three *Trichogramma* spp. in response to smaller scale habitat complexity in the form of small quantities of millet or flour (Grieshop et al. 2008), as well as their foraging response on retail shelving in the presence or absence of packaged goods (Grieshop et al. 2007b).

The three commercially available species selected for comparison were: *T. deion* (Pinto and Oatman), *T. ostriniae* Pang and Chen, and *T. pretiosum* (Riley). The commercial strains of *T. deion* and *T. pretiosum* used in this study were selected based on host preference tests performed by Schöller and Fields (2002). Their study demonstrated that these two species readily parasitized the eggs of *P. interpunctella* under both choice and no-choice conditions, while two strains of *T. minutum* Riley, and individual strains of *T. sibericum* Sorokina and *T. brassicae* Bezdenko did not. Furthermore, *T. pretiosum* has been collected from wild stored product moth populations in peanut warehouses (Brower 1984). *T. ostriniae* has been extensively explored as an augmentative biological control agent for *O. nubilalis* in field and sweet corn (Wang et al., 1997, 1999; Hoffman et al. 2002; Wright et al. 2002), was shown capable of parasitizing the eggs of *P. interpunctella* in bulk wheat (Jeffery Gardener, personal communication), and has a history of being reared on a similar stored product pyralid *Ephestia kuhniella* (Smith 1996). Likewise all three *Trichogramma* strains used in this study had been reared on *E. kuhniella* for more than 100 generations (Synthia Penn, personal communication; Jeffery Gardener, personal communication).

## Materials and Methods

### Insects

All insect species were maintained at the USDA-ARS Grain Marketing and Production Research Center in Manhattan, Kansas, USA, in a walk-in growth chamber set at 26 ± 1° C, 60 ± 5% RH, and a 16:8 light:dark photoperiod. Colonies of all three *Trichogramma* species were maintained on fresh (< 1 day old) eggs of *E. kuehniella* that had been sterilized by exposure to UV radiation. Unsterilized eggs of *P. interpunctella* were used in experiments. Both *P. interpunctella* and *E. kuehniella* were reared on a diet composed of cracked wheat, wheat shorts, honey, and glycerin (McGaughey and Beeman 1988). *T. deion, T. pretiosum*, and *E. kuehniella* starter cultures were provided by Beneficial Insectaries (www.insectary.com) in April 2003, March 2004, and February 2002, respectively. *T. ostriniae* were provided by Michael Hoffman's laboratory at Cornell University in November 2003. The *P. interpunctella* used in experiments had been in continuous culture at the Grain Marketing and Production Research Center for 10 or more years. Specimens of all species used in the experiment were deposited into the Kansas State University Museum of Entomological and Prairie Arthropod Research under voucher number 171.

Arrhenotokous strains of *T. deion* and *T. ostriniae* were used with observed sex ratios of 2:1 and 3:1 (female: male, respectively). Neither species was observed host feeding. The *T. pretiosum* strain was virtually thelytokous with an observed sex ratio of 24:1 (female: male), and was observed host feeding. Individual naïve, *Trichogramma* females were used in the trials. Females were less than 16 h old and had been allowed access to a 1:1 honey: water solution and mates for four hours prior to collection. Individual *Trichogramma* females were collected using a natural hair paintbrush to drive individual females up a small strip of paper (0.25 cm wide x 4 cm long) that was then placed in an empty 8-dram shell vial.

### Experimental design

A three by four nested factorial design consisting of three species and four combinations of intra- and interpatch densities was used. The four treatments consisted of: nine single-egg patches (three by three grid of single-egg patches spaced 15 cm apart); nine four-egg patches (a three by three grid of four-egg patches spaced 15 cm apart); 36 single-egg patches (a six by six grid of single-egg patches spaced 7.5 cm apart); and 36 four-egg patches (a six by six grid of four-egg patches spaced 7.5 cm apart).

Experimental trials consisted of testing a single species at all four of the intra- and interpatch treatment combinations. There were 15 trials (replications) for each species with each density and a single species represented in each trial. Each trial lasted six hours and was performed in a walk-in environmental growth chamber at 23 ± 1° C, 45 ± 5% RH, and under constant light conditions provided by four banks of three 40 W soft white 122 cm fluorescent tubes. The environmental settings used in the experiments were based upon temperature, humidity, and light conditions observed between April and November 2002, at four retail stores in Manhattan, KS, USA.

Treatment position on the lab bench was randomized for each experimental trial. Egg patches consisted of a 1 cm diameter cardstock disk with fresh (< 18 hr old) *P. interpunctella* eggs attached with a tragacanth paste, a nontoxic plant-derived glue (Merck, www.merck.com). In addition, 25–40 four-egg patches (100–160 eggs total) were kept in sealed Petri dishes for each trial to assess natural egg mortality unrelated to parasitoids. To avoid cross-contamination of *Trichogramma* cultures, trials with *T. deion* were run between June and November 2003; trials with *T. ostriniae* were run between January and June 2004; and, for *T. pretiosum,* they were run between June and August 2004. Thus, only a single species of *Trichogramma* was maintained in the laboratory colony at any one time. Treatments were run in separate Plexiglas covered arenas measuring 50 x 50 x 10 cm (L x W x H) that were open on one of the 50 x 50 cm sides to form the floor of the arenas. Sentinel egg patch grids were laid out on a fresh piece of butcher paper, and a single naïve female *Trichogramma* was placed in the center of a randomly selected central quadrat. Immediately following release of the female, the Plexiglas cover was centered and placed over the grid of sentinel egg patches. An airtight seal between the edges of the arena and the paper was maintained using a strip of adhesive weather-stripping. Plexiglas covers were cleaned with distilled water prior to and immediately after experimental runs to minimize static charge.

### Data collection

*Trichogramma* females were removed from arenas after six hours. Egg patches were numbered and placed in sealed Petri dishes, which were kept in a growth chamber set at 26 ± 1° C and 60 ± 7% RH. Host-egg condition was evaluated after seven days using a stereoscope (110–140x magnification). Eggs from individual egg patches were graded as hatched, parasitized, or dead; parasitized eggs were identified by their dark appearance and/or the presence of red pupal eyespots. The control egg patches were also judged at this time. All females used in the trials survived to the end of the experiment. Experimental runs with over 20% natural egg mortality were removed from the study, with additional runs made to bring the total number of replications per species to 15. A total of three, two, and two trials were repeated for *T. deion, T. pretiosum*, and *T. ostriniae*, respectively.

### Data analysis

Egg patches and egg parasitization data were analyzed using the mixed procedure (SAS 2001). Replicates with zero parasitism were removed from the analysis to reduce variability, resulting in 15, 13, and 14 replicates for *T. deion, T. pretiosum*, and *T. ostriniae*, respectively. The numbers of egg patches and eggs parasitized were analyzed in a three by four mixed model ANOVA with fixed factors consisting of species and the four treatments, and with species nested within experimental runs as a random factor. In addition, four three-way mixed model ANOVAs were run to test for differences among species within treatments, with species used as the fixed factor and species nested within experimental runs as the random factor. Three four-way ANOVAs, one for each species, were run for both the number of egg patches and eggs parasitized. Degrees of freedom were adjusted using the Kenward-Rodgers correction for mixed models with nesting, and multiple comparisons were run for all models using a Tukey-Kramer adjusted LSD (p = 0.05). Additional paired *t*-tests were run for each species comparing the number of egg patches parasitized for the nine single-egg patches treatment with the nine four-egg patches treatment, as well as the 36 single-egg patches treatment with the 36 four-egg patches treatment. Similar, paired *t*-tests were run for the number of eggs parasitized for the 36 single-egg patches treatment with the nine four-egg patches treatment. To assess whether there were differences in the number of non-responsive females among the three species and four treatments a two-way χ^2^ test was performed.

In addition to patch use and parasitism, the landscape class metric “normalized aggregation index” (nAI) was calculated for egg patches parasitized by each species in the 36 single-egg patches treatment and the 36 four-egg patches treatment. The nAI equals the total length of edge (or perimeter) involving the corresponding class, given in number of cell surfaces, minus the minimum length of class edge (or perimeter) possible for a maximally aggregated class, also given in number of cell surfaces, which is achieved when the class is maximally clumped into a single, compact patch, divided by the maximum minus the minimum length of class edge (McGarigal et al. 2002). The nAI has a range from zero to one, with a value of zero indicating a maximally aggregated class and a value of one indicating a maximally dispersed class.

For the purpose of nAI calculation, patches were treated as 7.5 x 7.5 cm cells in a 6 x 6 landscape array with two class values denoting either a parasitized patch or a patch lacking parasitism. nAI was calculated using the Fragstats software package (McGarigal et al. 2002) using the equation:

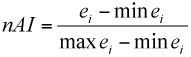

Where *e_i_* equals the actual total length of edge (or perimeter) of class *i*, min *e*_i_ equals the minimum possible total length of edge of class *i* based on the number of cells in class *i*, and max *e*_i_ equals the maximum possible total length of edge of class *i* based on the number of cells in class *i*. nAI values for the two treatments were analyzed for each of the three species using a paired *t*-test. Values were transformed using an angular transformation (arcsin(y^0.5^)) to stabilize variance.

## Results

### Egg patches parasitized

**Figure 1.**
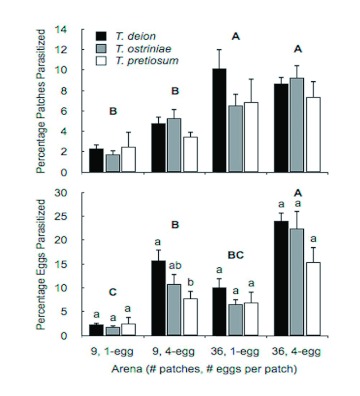
Mean (+SEM) patches and eggs parasitized by species and treatment. Bars with different lowercase letters over them were found to be significantly different among species within the same treatment, as were whole treatments with different uppercase letters (Tukey-Kramer adjusted LSD with p < 0.05). High quality figures are available online.

Significant differences were detected in the number of egg patches parasitized for treatment (*F* = 11.99; df = 3, 83.7; p = 0.0001), but not for species or for the species by treatment interaction (*F* = 0.22; df = 2, 1; p = 0.8358 and *F* = 0.98; df = 6, 83.6; p = 0.4408, respectively) (Figure 1). The number of patches parasitized was numerically highest in the 36 single-egg patches for *T. deion* (10.11 patches) and in the 36 four-egg patches for both *T. ostriniae* and *T. pretiosum* (9.27 and 7.36 patches, respectively). The number of patches parasitized was lowest in the nine single-egg patches for all three species (Table 1). *T. deion* parasitized the most egg patches in the 36 four-egg patch treatment and the fewest in the two nine-patch treatments. Significant differences for *T. ostriniae* were detected between the 36 four-egg patches and all three of the other treatments. No significant differences were detected in the number of patches parasitized among treatments for *T. pretiosum* (Table 1).

Both *T. deion* and *T. ostriniae* parasitized significantly more patches in the nine four-egg patches treatment compared with the nine single-egg patches treatment (Table 2). However, only *T. ostriniae* parasitized significantly more patches in the 36 four-egg patches treatment compared to the 36 single-egg patches treatment. No differences were observed for *T. pretiosum* regardless of patch size.

### Eggs parasitized

Significant differences were detected in the number of host eggs parasitized for treatment (*F* = 33.8; df = 3, 85; *P* = 0.0001) but not for species or species × treatment interaction (*F* = 0.9; df = 6, 85; p = 0.4990). However, species was nearly a significant factor (*F* = 2.79; df = 2, 83.7; p = 0.067) (Fig. 1). All three species parasitized the most eggs in the 36 four-egg patches treatment and the least number of eggs in the nine single-egg patches treatment. One-way ANOVA showed that *T. deion* parasitized significantly more eggs than *T. pretiosum* within the nine four-egg patches treatment (*F* = 3.67; df = 2, 21; p = 0.0429) (Figure 1).

With respect to patch effects on individual species, *T. deion* parasitized the most host eggs in the 36 four-egg patch treatments and the least in the nine single-egg patch treatment (Table 1). The trend in parasitism was similar for the other two *Trichogramma* species except that parasitism in the nine single-egg patch treatment was lowest, although not significantly so (Table 1).

To test whether the number of eggs parasitized was influenced by the total number of host eggs available versus the spatial pattern of host eggs (inter- and intra-patch configuration), parasitism for each species was compared for the two treatments containing 36 eggs (nine four-egg patches versus 36 single egg patches). No significant differences were detected for any of the species, although the trends for *T. deion* and *T. pretiosum* were nearly significant (p = 0.0914 and p = 0.0738, respectively) with trends for more eggs parasitized in the nine four-egg patches treatment than in the 36 single-egg patches treatment (Table 2).

### Number of non-responding females

A similar pattern of non-responsive females was found among the three species with the highest number of non-responsive females found in the nine single-egg patches treatment and the lowest number found in the 36 four-egg patches treatment (*X*^2^= 1.80, df = 3, *p* = 0.62). The exception was *T. ostriniae*, where the lowest number of non-responsive females was found in the 36 single-egg patches treatment (Table 3).

### Aggregation of parasitism

**Figure 2.**
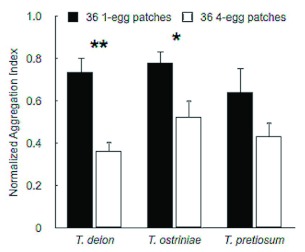
Mean (+SEM) normalized aggregation index for one-egg-36 patch and four-egg-36 patch arenas. Values closer to zero indicate a higher level of aggregation. Pairs of bars with stars over them are significantly different (Paired t-test ± < 0.05, ** < 0.01). High quality figures are available online.

Aggregation of parasitism was significantly greater in the 36 four-egg patch treatment than in the 36 single-egg patch treatment for both *T. deion* (*t* = 4.04; df = 20; p < 0.001) and *T. ostriniae* (*t* = 2.34; df = 17; p < 0.03). While a significant difference was not detected for *T. pretiosum*, nAI was slightly lower for the 36 four-egg patch treatment (Figure 2).

## Discussion

Although patterns of parasitism varied considerably among species, no one species performed consistently better than the other two. The fact that all three species parasitized the most eggs where egg density was highest (the 36 four-egg patches treatment), and the fewest where host density was lowest (the nine single-egg patches treatment) suggests that host-finding occurred in a density-dependent manner (Figure 1). The pattern of density-dependent parasitism is consistent with observations reported for *Trichogramma* under laboratory (Kfir 1983) and field conditions (Wang and Ferro 1998). For *T. deion* and *T. ostriniae*, the number of host patches parasitized was highest when interpatch distance was lowest (36 patch treatments) (Table 1). Other authors have attributed increased host patch discovery to increased intrapatch density, both in the laboratory (Barnay et al. 1999), and in the field (Morrison et al. 1980; McCravey and Berisford 1998).

**Table 1.**
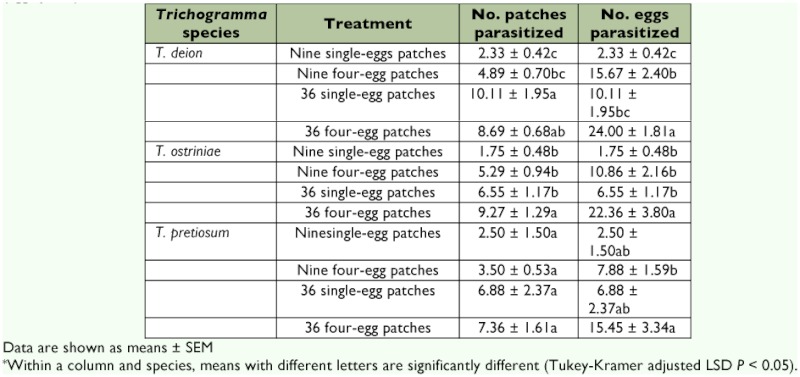
Mean ± SEM number of patches parasitized and number of eggs parasitized by *Trichogramma* species and treatment (eggs/patch).*

**Table 2.**
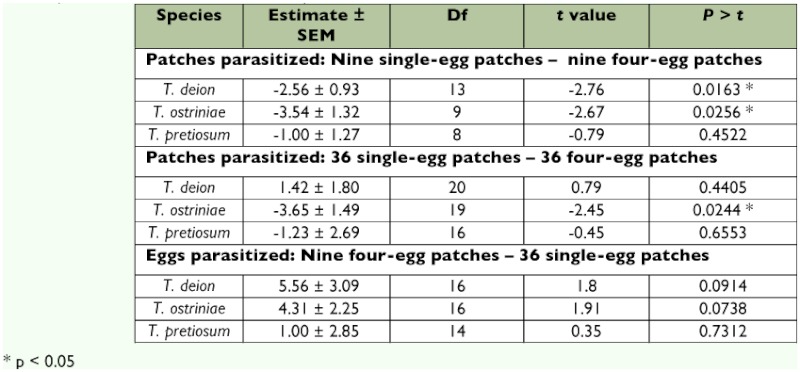
Paired *t*-test results for number of patches parasitized and number of eggs parasitized by trichogrammid species in test arenas (differences between means tested).

**Table 3.**
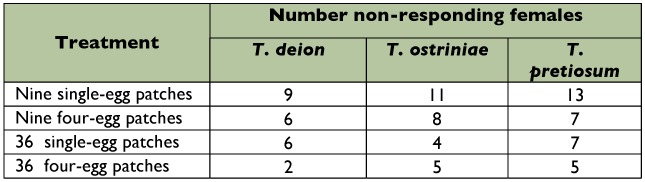
Number of replicates by trichogrammid species and patch size treatment with non-responding females (out of 15).

The three species responded differently in intra- and inter-patch comparisons. For *T. deion*, the number of patches parasitized was about two times greater in the nine four-egg patches treatment compared to the nine single-egg patches treatment. However, patches parasitized were similar in the 36 four-egg and single-egg patches treatments (Table 1). In contrast, *T. ostriniae* parasitized significantly more four-egg patches in both nine and 36 patch arrangements, and there were no intrapatch differences for *T. pretiosum* (Table 2). One possible explanation for the differences in response to host densities among species is that the three species perceive “patch structure” differently. The greater spatial aggregation of patches parasitized in the 36 four-egg patches treatment compared with the nine single-egg patches treatment for both *T. deion* and *T. ostriniae* could be due to a foraging response to “meta” or super patches consisting of more than a single disk when four-eggs were present (Figure 2). In contrast, the lack of difference for *T. pretiosum* suggests that *T. pretiosum* may have a tendency to respond to the egg patches as individual patches irrespective of intrapatch density. The difference between *T. pretiosum* and the other two *Trichogramma* species may be the result of the latter two species exhibiting an area-restricted search in response to the discovery of patches containing more hosts. For *T. ostriniae* this may be due to an evolutionary association with a host that lays dense egg masses versus sparsely distributed eggs.

An alternative hypothesis that may explain increased patch discovery as interpatch density increases is that host patch discovery depends on the detection of a minimum level of kairomones associated with the host. Although untested in this study, the host eggs may have produced volatile compounds identifiable by *Trichogramma* and used as host-foraging cues since other studies have shown that *Trichogramma* responds to kairomones produced by or associated with host eggs, including scales from adult moths, sex pheromones, and compounds associated with egg metabolic processes (Jones et al. 1973; Morrison and Nordlund 1980; Nordlund 1981; Boo and Yang 2000). In addition, it has been hypothesized that *Trichogramma* spp. require a minimum amount of stimulus prior to engaging in normal foraging behavior (Resznik and Umarova 1991). Given that a greater number of non-responsive females were consistently found at the lowest egg density is in general agreement with this hypothesis (Table 3). Further evidence in support of this hypothesis is the nearly significant trend of a greater number of eggs parasitized by *T. deion* and *T. ostriniae* for the nine four-egg patches treatment compared to the 36 single-egg patches treatment (p = 0.0914 and p = 0.0738, respectively) (Table 1). There may be different thresholds for density-dependent host-foraging responses in *Trichogramma*. For example, *T. ostriniae* exhibited greater parasitism only at the highest of the four host densities (Table 1).

The host densities and arrangements tested in this experiment were chosen because they provided an approximation of how *P. interpunctella* distributes eggs in warehouses and stores. A study by Arbogast and Mullen (1978) suggests that this distribution consists of single or small groups of eggs (< 10) per local group aggregated around a compromised package or food product spill. An experiment performed by Grieshop et al. (2006) testing *T. deion's* ability to manage *P. interpunctella* in small arenas containing either bulk corn meal or cornmeal in compromised packaging clearly indicated that the parasitoid was largely ineffectual at reducing *P. interpunctella* populations on bulk product, but reduced it by more than 75% in packaged products. Thus, trichogrammatids that respond to low densities of hosts and thoroughly search in areas where low numbers of eggs are found (i.e., on packages or adjacent shelf space,) would theoretically be better suited to manage this important stored product pest. In this case, both *T. deion* and *T. ostriniae* demonstrated a more aggregated search pattern at the highest host density, suggesting that they may perform better when foraging in finished stored product environments (Figure 2).

Two other experiments using the same three species, but conducted at different spatial scales, support the idea that *T. deion* is the best-suited species for biological control of *P. interpunctella*. The first was conducted at a smaller scale and examined the impact of microhabitat complexity on single females (Grieshop et al. 2008), while the second was conducted at a much larger scale and examined the foraging efficacy of mass-released parasitoids on retail shelving units (Grieshop et al. 2007). The failure of *T. deion* to outperform the other two species in this experiment may be due to either: 1) the difference in spatial scale among the present and previously published experiments, or 2) the homogeneous nature of the arenas used in this trial. Thorpe and Dively (1985) demonstrated that three species of *Trichogramma* responded differently depending on the size of arena used in laboratory tests. In these trials, two species of *Trichogramma* performed best at a smaller scale, while the third performed best at the largest spatial scale observed. Alternatively, multiple investigators have shown that habitat complexity affects *Trichogramma* foraging success in the laboratory (Andow and Prokrym 1990; Lukianchuk and Smith 1997; Gingras and Boivin 2002; Gingras et al. 2002, 2003; Andow and Olson 2003). Thus, it is possible that physical/structural homogeneity of the arenas used in this trial may have predisposed all three species to parasitize more hosts than when they were in a more heterogeneous habitat.

Although not entirely conclusive, these results suggest that *T. deion* is the best-suited species for biological control of *P. interpunctella. T. deion* parasitized slightly more host eggs than the other two species in all four treatments and significantly more than *T. pretiosum* in the 36 four-egg patches treatment (Figure 1). Furthermore, while intrapatch egg density appeared to increase patch discovery for *T. ostriniae* at the greater interpatch density, *T. deion* did not appear to be affected by this factor. Because the presence of a single *P. interpunctella* larva, pupa, or adult can result in the total economic loss of the product (Subramanyam et al. 2001), location of individual host eggs is of critical importance for *Trichogramma* used in retail stores or warehouses. Other studies comparing the three species' performance at either a finer spatial scale in response to microhabitat complexity or at a broader spatial scale examining the ability of mass-released *Trichogramma* to locate eggs on shelving units found that *T. deion* parasitized/killed significantly more *P. interpunctella* eggs than the other two species (Grieshop et al. 2007, Grieshop et al. 2008).

## Abbreviations

nAI: normalized aggregation index
